# Gut Microbiota Predicts the Risk of Future COVID-19 Hospitalization and Mortality: Insights From the Population-Based HELIUS Study

**DOI:** 10.1093/infdis/jiaf541

**Published:** 2025-10-31

**Authors:** Robert F J Kullberg, Brent Appelman, Henrike Galenkamp, Maria Prins, Bert-Jan van den Born, Max Nieuwdorp, Bastiaan W Haak, W Joost Wiersinga

**Affiliations:** Center for Infection and Molecular Medicine; Center for Infection and Molecular Medicine; Department of Public and Occupational Health, Amsterdam UMC, University of Amsterdam; Department of Infectious Diseases, Public Health Service of Amsterdam; Division of Infectious Diseases; Department of Public and Occupational Health, Amsterdam UMC, University of Amsterdam; Division of Vascular Medicine, Department of Internal Medicine, Amsterdam UMC, University of Amsterdam, the Netherlands; Division of Vascular Medicine, Department of Internal Medicine, Amsterdam UMC, University of Amsterdam, the Netherlands; Center for Infection and Molecular Medicine; Center for Infection and Molecular Medicine; Division of Infectious Diseases

**Keywords:** anaerobic bacteria, butyrate, COVID-19, gut microbiota, SARS-CoV-2

## Abstract

Gut microbiota are disrupted in patients hospitalized for COVID-19, with a loss of anaerobic bacteria-producing butyrate. Yet, these disruptions could either be a consequence of the infection itself or increase susceptibility from the outset. Here, we investigated whether gut microbiota influence the risk of future COVID-19 hospitalization and mortality. In 5084 participants of a population-based cohort, gut microbiota composition was associated with the risk of future severe COVID-19. Specifically, increased abundances of butyrate-producing bacteria were associated with a lower risk of severe COVID-19. Together, gut microbiota alterations precede severe COVID-19 and may represent a novel target for prevention.

## Teaser

In this large population-based cohort, we found that baseline gut microbiota, especially low levels of butyrate producers, are coupled with a higher risk of hospitalization or mortality due to COVID-19 during follow-up, even after adjustment for potential confounders.

Gut microbiota are extensively disrupted in patients hospitalized for COVID-19, with a loss of anaerobic bacteria producing the short-chain fatty acid butyrate [1, 2]. While traditionally viewed as a consequence of medical interventions and acute illness (eg, antibiotics, reduced food intake), an alternative explanation has emerged: gut microbiome disruptions may precede infection onset, exacerbate severity, and increase the likelihood of hospitalization. This paradigm shift is supported by preclinical studies demonstrating that obligate anaerobic bacteria strengthen host defenses against viral pneumonia, including COVID-19, through the production of the immunomodulatory metabolite butyrate [3–5]. In murine models of pneumonia, butyrate enhances the functioning of CD8+ T cells and alveolar macrophages while limiting hyperinflammation and tissue damage [4–6]. Yet, whether these preclinical findings translate to humans is unclear. We recently showed that high intestinal levels of butyrate-producing bacteria were associated with a reduced risk of future hospitalizations for infections of any type [7]. However, this relationship with all-cause infections may not explicitly apply to viral pneumonia, including SARS-CoV-2. Here, we investigated whether gut microbiota, specifically low amounts of butyrate-producing bacteria, precede severe COVID-19 and influence the risk of hospitalization in humans.

## METHODS

We used data from the large-scale, population-based Healthy Life in an Urban Setting (HELIUS) study. Details on study design have been published [7]. Briefly, HELIUS is a multiethnic, population-based, prospective cohort study conducted in Amsterdam, the Netherlands. Adults (aged 18–70 years at inclusion) were randomly sampled from the municipality register, stratified by ethnicity, and invited to participate. Participants completed questionnaires, underwent a physical examination, and provided fecal samples at inclusion (May 2011–November 2015). Fecal microbiota were profiled by 16S rRNA gene sequencing (V4 region) and subsequently preprocessed in Deblur (version 1.1.1) and Greengenes2 (release 2022.10), as previously described [7]. Ethical approval was obtained from the Academic Medical Center Ethical Review Board (protocol 10/100; amendment 10/100#10.17.1729; NL32251.018.10), and all participants provided written informed consent.

Our primary outcome was severe COVID-19, defined as hospitalization or mortality. Outcome data were retrieved from national registries (Statistics Netherlands) [7]. We focused on events during the first 2 years of the pandemic (between 1 January 2020 and 31 December 2021), given the widespread vaccination uptake afterward and to limit the effects of differing pathogenicity of circulating SARS-CoV-2 variants (eg, patients hospitalized with Omicron are older, of different ethnic backgrounds, and have more comorbidities) [8].

We assessed associations between our outcome and 3 predefined gut microbiota features: community composition (β-diversity and individual genera), community diversity, and the cumulative relative abundance of key butyrate-producing bacteria (given the loss of butyrate producers in hospitalized patients with COVID-19 and the protective effects of butyrate in animal experiments) [1, 2, 4–6]. Butyrate-producing taxa were categorized as previously described, which accurately predicts fecal butyrate concentrations (Pearson *r* = 0.76) [7, 9, 10]. Time-to-event analyses with Cox proportional hazards models were used to examine the relationship between the primary outcome and individual genera (restricted to genera with a minimum abundance of 0.005% in at least 10% of participants), microbiota diversity, or butyrate-producing bacteria. Relative abundances were centered log ratio transformed to correct for the inherent compositional nature of microbiome data. To adjust for potential confounders, multivariable models included baseline age, sex, ethnicity, body mass index, time between baseline and 1 January 2020, and comorbidities (hypertension, diabetes, and cancer, as well as cardiovascular, pulmonary, and gastrointestinal disease). Additionally, we used nationwide SARS-CoV-2 testing data to compare the microbiota of participants with severe COVID-19 against those with mild to moderate SARS-CoV-2 infection (ie, positive polymerase chain reaction result not resulting in hospitalization or mortality). The χ^2^ test was used to compare categorical variables. Continuous variables were compared with Wilcoxon rank sum tests. Two-tailed level of significance was set at *P* < .05.

## RESULTS

In total, 5084 HELIUS participants had gut microbiota sequenced, consented for linkage with national registries, and were alive at 1 January 2020. During follow-up, 73 participants reached the primary outcome (COVID-19–related hospitalization or mortality). Participant characteristics are depicted in Table 1. Characteristics subset by several clinical and microbiota features are provided in the supplementary material.

**Table 1.** Characteristics of the Study Cohort

	Median (IQR) or No. (%)		
	Severe COVID-19 (n = 73)^a^	No Severe COVID-19 (n = 5011)	*P* Value
Age, y	57 (48–62)	52 (43–59)	<.001
Female sex	28 (38.4)	2618 (52.2)	.025
Ethnicity			<.001
Dutch	2 (2.7)	1503 (30.0)	
African Surinamese	21 (28.8)	1190 (23.7)	
South-Asian Surinamese	27 (37.0)	792 (15.8)	
Turkish	11 (15.1)	437 (8.7)	
Moroccan	9 (12.3)	587 (11.7)	
Ghanaian and other	3 (4.1)	502 (10.0)	
Body mass index	30.5 (27.8–33.9)	26.5 (23.8–30.0)	<.001
Current or former smoker	41 (56.9)	2579 (52.2)	.49
Comorbidities			
Hypertension	35 (49.3)	1274 (25.7)	<.001
Diabetes	22 (30.6)	526 (10.6)	<.001
Cardiovascular disease	16 (22.5)	488 (9.9)	.001
Pulmonary disease	19 (26.4)	505 (10.2)	<.001
Gastrointestinal disease	9 (12.5)	385 (7.8)	.21
Cancer	1 (1.4)	128 (2.6)	.79
Registered positive SARS-CoV-2 PCR result^a^	73 (100)	861 (17.2)	

Abbreviation: PCR, polymerase chain reaction.

^a^Outcomes were assessed during the first 2 years of the COVID-19 pandemic (1 January 2020–31 December 2021).

**Figure 1.**
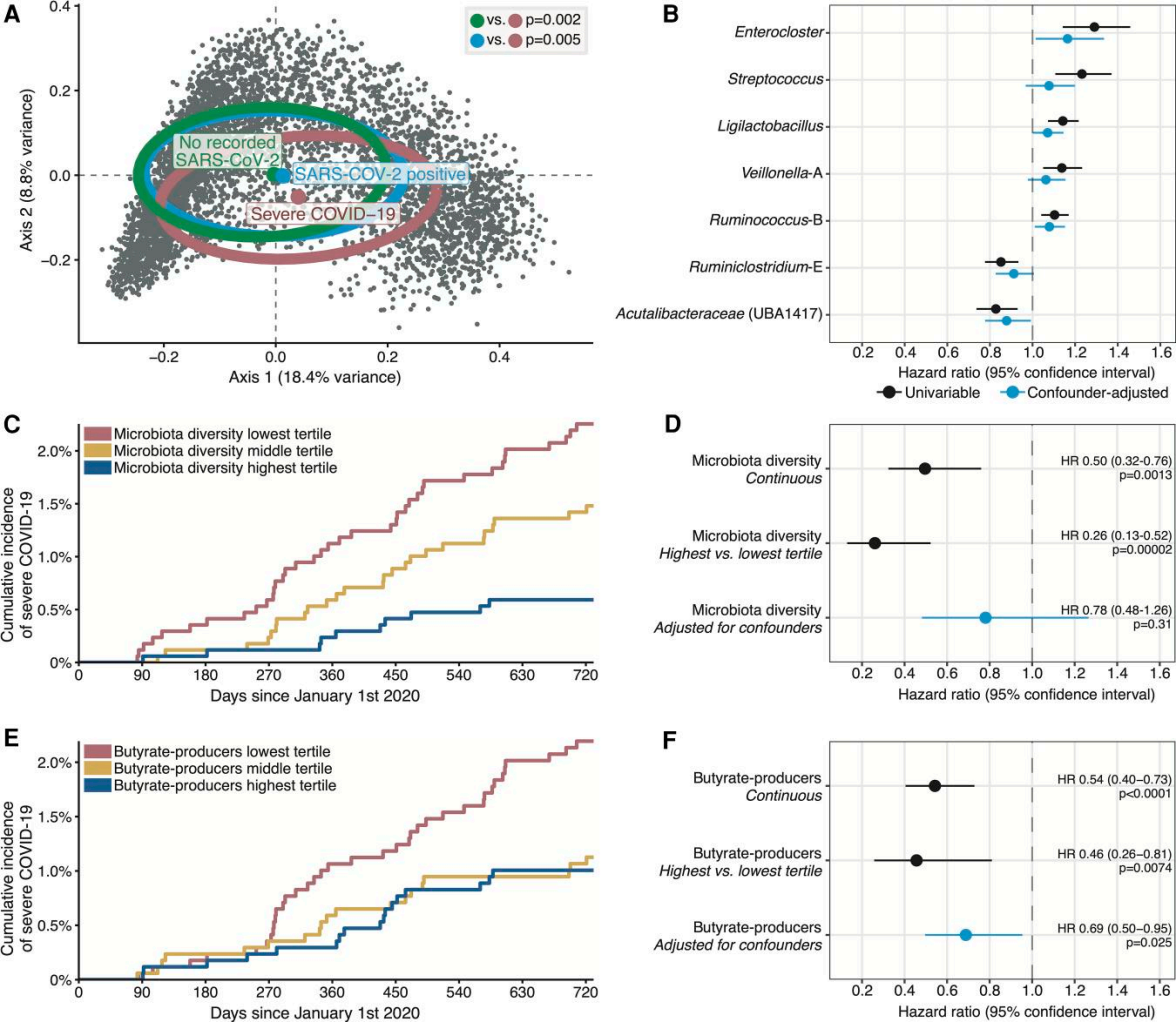
Gut microbiota are associated with the risk of future severe COVID-19. *A*, Baseline gut microbiota composition differed between participants with future severe COVID-19 (hospitalization or mortality, n = 73) and participants without severe COVID-19 during follow-up (those with a registered positive PCR result for SARS-CoV-2, n = 861; those with no recorded positive SARS-CoV-2 PCR, n = 4150). Permutational multivariate analysis of variance with Bray-Curtis dissimilarities at the species level were used to assess differences between groups. Smaller points represent individual participants. Larger points depict the group centers and ellipses the 50% confidence interval. *B*, Higher relative abundances of *Enterocloster* and several facultative anaerobic bacteria (eg, *Streptococcus*, *Limosilactobacillus*, *Ligilactobacillus*) were associated with an increased risk of severe COVID-19, whereas several members of the Oscillospirales order (eg, *Ruminiclostridium-E*, Acutalibacteraceae UBA1417) were associated with a lower risk. Individual bacterial genera associated with the primary outcome were identified through Cox proportional hazards models. Significant univariable associations are shown (Benjamini-Hochberg adjusted *P* < .05). Confounder-corrected analyses were adjusted for age, sex, ethnicity, body mass index, time between inclusion and 1 January 2020, and comorbidities (hypertension, diabetes, and cancer, as well as cardiovascular, pulmonary, and gastrointestinal disease). *C*, Cumulative incidence of severe COVID-19, stratified by tertiles of gut microbiota diversity (Shannon index at the species level). Participants who died from causes other than COVID-19 were censored. Lower diversity was associated with an increased risk of the primary outcome. *D*, This was not significant after correction for potential confounders. *E*, Participants with higher relative abundances of butyrate-producing bacteria had a decreased risk of severe COVID-19 as compared with participants with lower abundances, when analyzed as continuous feature and when stratified by tertiles of butyrate producer abundance. *F*, The association between the relative abundance of butyrate-producing bacteria and severe COVID-19 remained significant when corrected for potential confounders in a multivariable Cox proportional hazards model.

The overall gut microbiota composition of participants who reached the primary outcome differed from participants without severe COVID-19 (Figure 1*A*). Higher relative abundances of the anaerobic gut symbiont *Enterocloster* were, among other genera, associated with an increased risk of future severe COVID-19, whereas members of the butyrate-producing Oscillospirales order were associated with a lower risk (Figure 1*B*). Higher gut microbiota diversity was associated with a reduced risk of severe COVID-19 (Figure 1*C*). However, this was not significant when adjusted for potential confounders (age, sex, ethnicity, body mass index, time between inclusion and 1 January 2020, and comorbidities; Figure 1*D*). In line with the protective effects in mice [4, 5], increased relative abundances of butyrate-producing bacteria were associated with a lower risk of severe COVID-19 when analyzed as a continuous variable and when comparing the lowest and highest tertiles (Figure 1*E* and 1*F*). The association between butyrate-producing bacteria and a reduced risk of severe COVID-19 remained when corrected for potential confounders in the multivariable model (Figure 1*F*). Associations between characteristics of our participants and the abundance of butyrate-producing bacteria have been described [7]. Three sensitivity analyses yielded similar results. First, we added antibiotic exposure in the 3 months prior to sample collection as a potential confounder to our multivariable model. Second, we focused solely on events in 2020. Third, participants were censored at the date of their first SARS-CoV-2 vaccination.

Finally, we restricted our analysis to participants with registered SARS-CoV-2 infection during follow-up (n = 934). Among these participants, gut microbiota differed between those with and without severe COVID-19 (*P* = .005; Figure 1*A*). The abundance of butyrate producers was lower in participants with severe COVID-19 as compared with participants with mild SARS-CoV-2 infection (no hospitalization or mortality; Wilcoxon rank sum test, *P* = .0099; data not shown).

## DISCUSSION

In this large population-based cohort, we found that gut microbiota are associated with the risk of future severe COVID-19. Baseline gut microbiota composition differed between participants with and without hospitalization or mortality due to COVID-19 during follow-up. Specifically, high amounts of butyrate-producing bacteria were associated with a reduced risk of future severe COVID-19, even after adjustment for potential confounders.

Animal experiments showed protective effects of the microbiota-derived immunomodulatory metabolite butyrate against viral pneumonia. Butyrate enhances epithelial barrier function and prevents translocation of pathogens from the gut into the bloodstream [3, 6, 11]. In addition, butyrate influences systemic and pulmonary immune responses through the activation of G protein–coupled receptor signaling and inhibition of histone deacetylase activity, resulting in increased CD8^+^ T cell–intrinsic antiviral responses and reduced airway neutrophilia, thereby limiting lung damage [4, 6]. Our findings align with these experiments, translating the preclinical evidence to humans. Moreover, we expand on previous studies showing that colonization with butyrate producers is associated with protection against infections in hematopoietic stem cell transplant recipients and the general population [7, 9]. Here, we found that the role of butyrate-producing gut microbiota is not limited to high-risk groups or all-cause infections but also applies to viral pneumonia. Combined with the preclinical evidence, our findings suggest that microbiome-directed therapies aimed at increasing intestinal abundances of butyrate producers might limit the risk of future severe viral pneumonia. For example, recent murine studies showed that the delivery of butyrate-producing bacteria (eg, *Faecalibacterium prausnitzii* or other Oscillospiraceae) dampened inflammation and improved the outcomes of bacterial and viral pneumonia—motivating studies investigating the clinical potential of such novel probiotics [5, 12, 13].

Among participants with a documented positive SARS-CoV-2 polymerase chain reaction result, gut microbiota differed between those with and without severe COVID-19, with a loss of butyrate-producing bacteria associated with severe COVID-19. Contracting SARS-CoV-2 is probably mainly driven by exposure to the virus, while gut microbiota are linked with development of severe disease following infection (we did not examine associations between microbiota and the risk of acquiring SARS-CoV-2). This work is limited by the time between sample collection and the start of the COVID-19 pandemic. Microbiota probably changed over time, which may obscure the relationship with outcomes, although microbiota reportedly are stable for years [14]. Moreover, although previous studies showed that relative abundances of butyrate-producing bacteria strongly correlate with actual fecal butyrate concentrations [7, 9, 10], butyrate production depends on strain-level differences in gut bacteria and environmental context, which we did not account for here.

In conclusion, we showed that gut microbiota, especially low levels of butyrate producers, are coupled with a higher risk of severe COVID-19. Butyrate-producing gut microbiota may represent a novel therapeutic target for the prevention of viral pneumonia.

## Supplementary Material

jiaf541_Supplementary_Data

## Supplementary Data

Supplementary materials are available at *The Journal of Infectious Diseases* online (http://jid.oxfordjournals.org/). Supplementary materials consist of data provided by the author that are published to benefit the reader. The posted materials are not copyedited. The contents of all supplementary data are the sole responsibility of the authors. Questions or messages regarding errors should be addressed to the author.
