# Supplementary material for: Gut Microbiota Predicts the Risk of Future COVID-19 Hospitalization and Mortality: Insights From the Population-Based HELIUS Study
Source: J Infect Dis. 2025 Oct 31;233(3):e823–7. doi: 10.1093/infdis/jiaf541 (PMC13017446; doi:10.1093/infdis/jiaf541)
Supplement: jiaf541_Supplementary_Data [file jiaf541_supplementary_data.pdf]

Supplementary Material

Gut microbiota predicts the risk of future COVID-19 hospitalization and mortality: insights from the population-based HELIUS Study

Robert F.J. Kullberg, Brent Appelman, Henrike Galenkamp, Maria Prins, Bert-Jan van den Born, Max Nieuwdorp, Bastiaan W. Haak, W. Joost Wiersinga

Supplementary Table 1

	Registered positive SARS-CoV-2 PCR[1] (n=934)	No registered positive SARS-CoV-2 PCR[1] (n=4150)	P value
**Age (median, IQR)**	49 (41-56)	52 (43-59)	<0.001
**Female sex**	487 (52.1)	2159 (52.0)	0.98
**Ethnicity**			<0.001
Dutch	182 (19.5)	1323 (31.9)	
African Surinamese	231 (24.7)	980 (23.6)	
South-Asian Surinamese	175 (18.7)	644 (15.5)	
Turkish	117 (12.5)	331 (8.0)	
Moroccan	171 (18.3)	425 (10.2)	
Ghanaian and other	58 (6.2)	447 (10.8)	
**Body mass index (median, IQR)**	27.3 (24.5-30.4)	26.4 (23.7-29.9)	<0.001
**Current or former smoker**	522 (56.9)	2098 (51.2)	0.002
**Comorbidities**			
Hypertension	242 (26.4)	1067 (25.9)	0.80
Diabetes	107 (11.6)	441 (10.7)	0.47
Cardiovascular disease	102 (11.1)	402 (9.9)	0.27
Pulmonary disease	104 (11.3)	420 (10.2)	0.37
Gastrointestinal disease	70 (7.6)	324 (7.9)	0.82
Cancer	24 (2.6)	105 (2.6)	1.00

Supplementary Table 1. Characteristics of the study cohort subset by registered SARS-CoV-2 status

Data are number of participants (%) unless otherwise indicated. IQR=interquartile range.

[1] During the first two years of the COVID-19 pandemic (between January 1st 2020 and December 31st 2021)

Supplementary Table 2

	No antibiotics prior to sample collection[1] (n=4456)	Antibiotics prior to sample collection[1] (n=488)	P value
**Age (median, IQR)**	52 (43-59)	51 (44-59)	0.48
**Female sex**	2268 (50.9)	308 (63.1)	<0.001
**Ethnicity**			<0.001
Dutch	1366 (30.7)	103 (21.1)	
African Surinamese	1076 (24.1)	105 (21.5)	
South-Asian Surinamese	705 (15.8)	89 (18.2)	
Turkish	384 (8.6)	47 (9.6)	
Moroccan	504 (11.3)	77 (15.8)	
Ghanaian and other	421 (9.4)	67 (13.7)	
**Body mass index (median, IQR)**	26.5 (23.8-29.9)	27.9 (24.7-31.3)	<0.001
**Current or former smoker**	2276 (51.8)	276 (57.6)	0.017
**Comorbidities**			
Hypertension	1123 (25.5)	276 (57.6)	0.011
Diabetes	453 (10.3)	81 (16.9)	<0.001
Cardiovascular disease	427 (9.7)	58 (12.3)	0.088
Pulmonary disease	430 (9.7)	78 (16.3)	<0.001
Gastrointestinal disease	311 (7.1)	71 (14.9)	<0.001
Cancer	104 (2.4)	18 (3.8)	0.085

Supplementary Table 2. Characteristics of the study cohort subset by antibiotic exposure before faecal sample collection

Data are number of participants (%) unless otherwise indicated. IQR=interquartile range.

[1] Antibiotic exposure during the 3 months before faecal sample collection.

Supplementary Table 3

	Lowest tertile of Butyrate-producers (n=1695)	Highest tertile of butyrate-producers (n=1694)	P value
**Age (median, IQR)**	52 (43-59)	52 (41-59)	0.34
**Female sex**	869 (51.3)	877 (51.8)	0.78
**Ethnicity**			<0.001
Dutch	304 (17.9)	686 (40.5)	
African Surinamese	426 (25.1)	368 (21.7)	
South-Asian Surinamese	368 (21.7)	191 (11.3)	
Turkish	149 (8.8)	148 (8.7)	
Moroccan	257 (15.2)	157 (9.3)	
Ghanaian and other	191 (11.3)	144 (8.5)	
**Body mass index (median, IQR)**	27.1 (24.3-30.6)	26.2 (23.4-29.4)	<0.001
**Current or former smoker**	902 (54.2)	846 (50.4)	0.030
**Comorbidities**			
Hypertension	493 (29.5)	394 (23.5)	<0.001
Diabetes	278 (16.6)	122 (7.2)	<0.001
Cardiovascular disease	209 (12.6)	126 (7.5)	<0.001
Pulmonary disease	205 (12.3)	155 (9.2)	0.005
Gastrointestinal disease	157 (9.4)	111 (6.6)	0.003
Cancer	52 (3.1)	39 (2.3)	0.19
**Severe COVID-19[1]**	37 (2.2)	17 (1.0)	0.009

Supplementary Table 3. Characteristics of participants within the lowest *versus* highest tertile of butyrate-producing bacteria

Participants were divided into tertiles (low, intermediate and high) based on their cumulative relative abundance of key butyrate-producing bacteria. Data are number of participants (%) unless otherwise indicated. IQR=interquartile range.

[1] During the first two years of the COVID-19 pandemic (between January 1st 2020 and December 31st 2021)
